# Metabolic Homeostasis in Life as We Know It: Its Origin and Thermodynamic Basis

**DOI:** 10.3389/fphys.2021.658997

**Published:** 2021-04-23

**Authors:** David F. Wilson, Franz M. Matschinsky

**Affiliations:** Department of Biochemistry and Biophysics, Perelman School of Medicine, University of Pennsylvania, Philadelphia, PA, United States

**Keywords:** metabolic homeostasis, metabolic regulation, glycolysis, energy metabolism, pyruvate kinase

## Abstract

Living organisms require continuous input of energy for their existence. As a result, life as we know it is based on metabolic processes that extract energy from the environment and make it available to support life (energy metabolism). This metabolism is based on, and regulated by, the underlying thermodynamics. This is important because thermodynamic parameters are stable whereas kinetic parameters are highly variable. Thermodynamic control of metabolism is exerted through near equilibrium reactions that determine. (1) the concentrations of metabolic substrates for enzymes that catalyze irreversible steps and (2) the concentrations of small molecules (AMP, ADP, etc.) that regulate the activity of irreversible reactions in metabolic pathways. The result is a robust homeostatic set point (−ΔG_ATP_) with long term (virtually unlimited) stability. The rest of metabolism and its regulation is constrained to maintain this set point. Thermodynamic control is illustrated using the ATP producing part of glycolysis, glyceraldehyde-3-phosphate oxidation to pyruvate. Flux through the irreversible reaction, pyruvate kinase (PK), is primarily determined by the rate of ATP consumption. Change in the rate of ATP consumption causes mismatch between use and production of ATP. The resulting change in [ATP]/[ADP][Pi], through near equilibrium of the reactions preceding PK, alters the concentrations of ADP and phosphoenolpyruvate (PEP), the substrates for PK. The changes in ADP and PEP alter flux through PK appropriately for restoring equality of ATP production and consumption. These reactions appeared in the very earliest lifeforms and are hypothesized to have established the set point for energy metabolism. As evolution included more metabolic functions, additional layers of control were needed to integrate new functions into existing metabolism without changing the homeostatic set point. Addition of gluconeogenesis, for example, resulted in added regulation to PK activity to prevent futile cycling; PK needs to be turned off during gluconeogenesis because flux through the enzyme would waste energy (ATP), subtracting from net glucose synthesis and decreasing overall efficiency.

## Introduction

### Fundamental Metabolic Requirements for Development of Life

Life depends on continuous input of energy because living cells require continuous assembly, maintenance, and selective destruction (turnover) of complex structures. These include both molecular (RNA, DNA, and proteins, etc.) and physical structures (membranes, organelles, etc.), as well as maintenance of non-equilibrium distributions of small molecules and ions. Energy input is needed to overcome both the negative entropy associated with making and maintaining order and the positive free energy associated with synthesis of the many required molecules. Providing and maintaining a robust, stable source of energy for doing chemical and physical work is the first and most essential requirement for the existence of life. This energy source has to be able to provide energy “on demand” because variations in the environment result in variations in metabolic energy consumption. As a result, energy metabolism in living organisms has a common homeostatic set point supported by metabolism that is “fitted” to their particular environment (temperature, pH, light level, hydration, and available nutrients, etc.). Support metabolism makes use of fuels available in the environment to match energy (ATP) production to energy demand as imposed by that environment. In general, the more complex the organism the more stable the energy source has to be for survival. As a result, although all living organisms have a common homeostatic set point, higher organisms actually provide the most focused model for understanding homeostasis. With increasing metabolic complexity the requirements for metabolic integration suppress the amount of sustained deviation from the core set point that the cell/organism can tolerate. In the discussion that follows, it is important to keep in mind that: 1. In higher organisms, cellular [ATP] is held quite constant. Substantial changes in [ATP] are typically associated with extreme conditions and potentially pathological events; 2. Many of the reactions that consume ATP are not dependent on the concentration of ATP per se; and 3. The rate of production of ATP is tightly coupled to the rate of ATP consumption, i.e., the steady state rate of production of ATP is determined by the rate of ATP consumption. Note: these assumptions may not apply during metabolic transients or under metabolic conditions that are not sustainable. These include rest-work and work-rest transitions and heavy work in muscle, as well as non-physiological conditions (hypoxia, ischemia, etc.).

### Limitations in Current Understanding/Descriptions of Metabolic Control and Metabolic Homeostasis

Descriptions of metabolism typically focus on the individual metabolic pathways and discuss their regulation in terms of kinetic control by individual reactions within the pathway. Emphasis is placed on identifying “rate limiting steps” usually attributed to reactions in the pathway that are irreversible, i.e., do not have significant reverse reaction under physiological conditions. In the present paper, we argue that emphasizing kinetic control through altering the activity of enzymes catalyzing the irreversible reactions in a metabolic pathway can be very misleading and interferes with achieving a more global understanding of how metabolism functions. To support life, the myriad of metabolic pathways in each individual cell must function as a coherent whole. For individual cells to survive, each pathway needs to be coordinated with, and complementary to, every other pathway in order to form an integrated ensemble. Similarly, for a complex organism to survive, each cell and tissue must coordinate with all of the other cells and tissues. Within an organism many diverse cells and tissues not only work together but are produced by differentiation from a common progenitor cell. Moreover, organisms and their metabolism remain stable over many generations. The extraordinary metabolic stability and reproducibility of this complex whole is referred to as metabolic homeostasis. In the present paper, we argue that this stability is based on, and intrinsic to, energy metabolism and was established very early in the evolution of living organisms.

Life arose in what would seem a very hostile environment. The primordial atmosphere on earth 4.8 billion years ago (Gya) to about 3.5 Gya is thought to have been reducing, high in CO_2_ and N_2_ but without O_2_ (Catling and Claire, 2005; Sánchez-Baracaldo and Cardona, 2020). Significant levels of oxygen appeared only after early life forms developed oxygen producing photosynthesis (about 3.5 Gya) and began producing oxygen. Initially, the reducing environment assured that oxygen concentrations in the atmosphere remained low and limited to the local environment of the photosynthetic organisms. This continued until sufficient oxygen had been produced to consume the excess of reductants in the environment. The global transition from a reducing to an oxidizing environment lasted about 2 billion years, from 3 to about 1 Gya, and was associated with oxidation of soluble ferrous iron salts dissolved in the oceans. This produced insoluble ferric oxide (rust) which precipitated, staining ocean sediments a reddish color. Dating these iron oxide stained sediments provides a geological record of the time required for transition from a reducing to an oxidizing environment (“the great rusting event”). Once reductant levels in the oceans were sufficiently low, by about 1 Gya, atmospheric oxygen rose rapidly, in geological time, to slightly above current levels (>20%).

### Carbon Fixation and Early Metabolism

**FIGURE 1:**
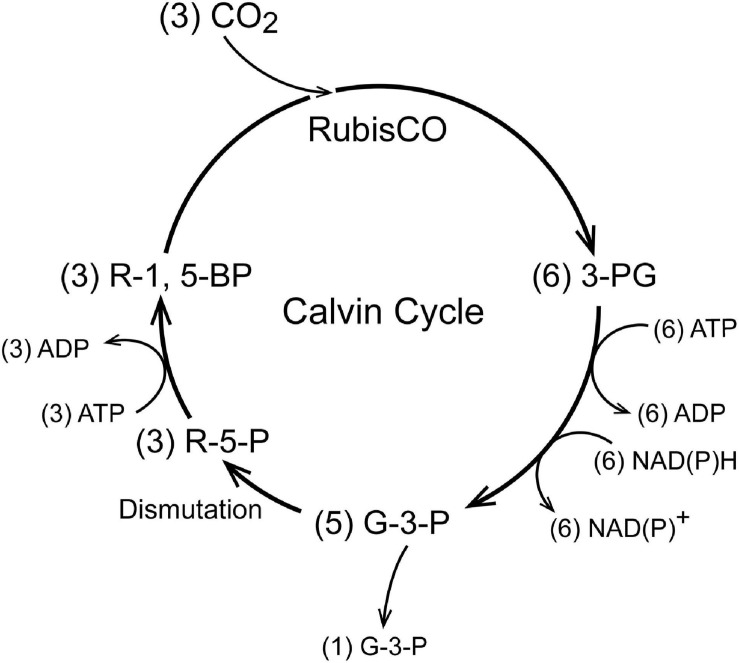
A schematic representation of carbon fixation by the Calvin Cycle. The enzyme ribulose-1,5-bisphosphate carboxylase (RubisCO) adds CO_2_ to ribulose-1,5-bisphosphate (R-1,5-BP) and splits the product into 2 molecules of 3-phosphoglycerate (3-PG). The 3-PG is then reduced to glyceraldehyde-3-phosphate which dismutates to regenerate the 5-carbon ribulose-5-phosphate (R-5-P). Phosphorylation of R-5-P regenerates R-1,5-BP for the next cycle. Only one carbon is added per turn of the cycle. Three turns of the cycle are required for a net increase of one G-3-P. The number of molecules involved in synthesis of one G-3-P is given in parentheses. Although a total of 6 G-3-P are formed, five of these are dismuted to produce the ribulose sugars required for maintaining cycle function.

The earliest life forms were autotrophs, using energy from photosynthesis or oxidation/reduction of reactive nitrogen or sulfur compounds in the environment to produce ATP and reductant [NAD(P)H] for synthesis of organic molecules from CO_2_ (carbon fixation). Whatever the energy source, in order to synthesize the many organic molecules needed to construct a living organism, carbon fixation was required. Carbon fixation is accomplished in plants, archaea, and bacteria by cyclic metabolic pathways in which an organic precursor molecule is carboxylated and then reduced, each turn of the cycle consuming one or more CO_2_ and increasing the organic carbon pool. Six different pathways for carbon fixation have been identified (Wächtershäuser, 1990; Berg, 2011; Fuchs, 2011). Of these, the Calvin Cycle, also called the Calvin-Benson or Calvin-Benson-Bassham Cycle, is the most prevalent and has been responsible for the greatest amount of carbon fixation. We will focus on the Calvin Cycle (Figure 1) in our discussion, but readers should keep in mind that it has not been established at which point in evolution the different pathways for carbon fixation appeared.

**FIGURE 2:**
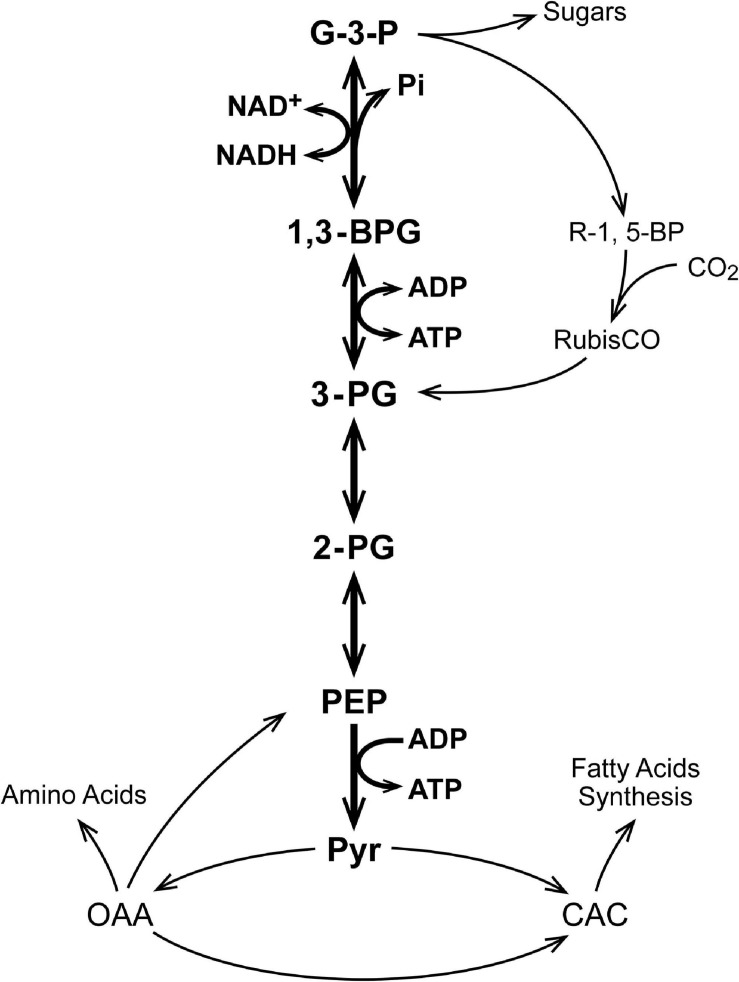
A schematic for operation of the Calvin Cycle in early autotropes. In green plants, the Calvin Cycle occurs within the chloroplast and uses NADPH as the reductant but this is a “recent” evolutionary development (1–2 Gya). These core reactions are coupled to all other metabolism in autotropes, in which all of the organic molecules in the organism are derived from G-3-P. Only a few of the coupled metabolic pathways are indicated.

As seen in Figure 1, in the Calvin Cycle ribulose-1,5-bisphosphate (R-1,5-BP, 5 carbons) is carboxylated and split into two molecules of 3-phosphoglycerate (3-P-G) by ribulose bisphosphate carboxylase (RubisCO; Tabita et al., 2008). The 3-P-G is then reduced to glyceraldehyde-3-phosphate (G-3-P) using NAD(P)H and ATP provided by photosynthetic phosphorylation. Most (5 out of 6) of the G-3-P molecules synthesized undergo dismutation to regenerate ribulose-1,5-bisphosphate, the substrate for RubisCO. One extra G-3-P is generated for each 3 turns of the cycle. In autotrophs with the Calvin Cycle, all of the organic molecules in the organism are synthesized from G-3-P. Although in modern (green plant) carbon fixation by the Calvin cycle the reduction is specific for NADPH and not NADH, it is unlikely that this nucleotide selectivity was present in early photosynthesis in prokaryotes and archaea. Reactions 1 and 2 (below) readily reduce 3-P-G to G-3-P when supplied with NADH and ATP. A metabolic extension of particular importance is oxidation of phosphoenolpyruvate (PEP) to pyruvate, another important biosynthetic precursor (Sauer and Eikmanns, 2005). In addition to pyruvate, oxidation of G-3-P through pyruvate kinase (PK) provides reducing equivalents (NADH) and ATP to support biosynthesis and energy metabolism when the photosynthetic production is insufficient and there is a source of G-3-P (Figure 2). G-3-P oxidation to pyruvate through a sequence of 5 reactions that became a critical part of glycolysis (the Embden-Meyerhof-Parnas pathway):

(1) $$\text{G-}\,3\,\text{-P}+\text{Pi}+{\text{NAD}}^{+}⟷1,3\,\text{-BPG}+\text{NADH}+{\text{H}}^{+}$$

(2) 1,3⁢-BPG+ADP⟷ 3⁢-PG+ATP

(3) 3⁢-PG⟷2⁢-PG

(4) 2⁢-PG⟷PEP

(5) PEP+ADP→Pyr+ATP

The pH (pH = 7.1, cytoplasm: pH = 7.4, and mitochondrial matrix) and [Mg^2+^] have been assumed constant and are not explicitly included in the reactions or reaction parameters. Enzymes catalyzing reactions 1–5 are present in branches of all living organisms (archaea, bacteria, and eukaryotes) and are highly conserved. Genetic analyses are consistent with their having originated early in the development of life (Fuchs, 1989; Ronimus and Morgan, 2003; Bräsen et al., 2014; Edirisinghe et al., 2016; Eme et al., 2017). We argue that the properties of reactions 1–5 and their metabolites may have played an important role in establishing the “metabolic core” on which living organisms, as we know them, are based. Reactions 1–4 are freely reversible and under physiological conditions near equilibrium (Veech et al., 1979). The physiological concentrations of the metabolites have been measured, equilibrium constants determined, and mass action ratios shown to equal, within experimental error, to the equilibrium constants. Because the equilibrium constants are thermodynamic parameters, we can be confident they have not changed since the origin of life.

Table 1 lists relevant physiological metabolite measurements made for erythrocytes (cells) as well as liver, brain, and skeletal muscle tissue of rats (Veech et al., 1979). The goal of these investigators was to determine the physiological value for the energy state {[ATP]/[ADP]_f_[Pi]} in different mammalian tissues. The concentration of ADP ([ADP]_f_) has a subscript f to show it is the free (unbound) concentration. ATP and Pi are present in mM concentrations in the cell cytoplasm. The cell/tissue content of ADP is only about 1/10 that of ATP and Pi, and ADP binds tightly to relatively abundant cellular proteins (Veech et al., 1979). The authors measured the metabolite ratios for three different near equilibrium reactions in which ADP was a reactant. They concluded that only about 5% of the ADP in tissues was free in solution and therefore contributed to the cytoplasmic energy state {[ATP]/[ADP]_f_[Pi]}. The energy state calculated using [ADP]_f_ was similar for all 4 tissues, near 3 × 10^4^ M^–1^. When the lower concentration of Mg^2+^ in erythrocytes compared to the other tissues (0.15 mM vs 1 mM) was taken into account, the calculated free energy of hydrolysis of ATP (ΔG_ATP_) was near −58.6 kJ/mole (−14 Kcal/mole) and not significantly different. Erythrocytes are, however, terminally differentiated, having lost their nucleus and nuclear content. For simplicity, erythrocytes will not be further discussed. Brain tissue is made up of many different cell types and is structurally complex. We have, therefore, chosen to focus our further discussion on liver and skeletal muscle. Although liver and skeletal muscle each contains several types of cells, most of the tissue is attributable to one, hepatocytes in liver and muscle cells in skeletal muscle.

**TABLE 1:** Metabolites relevant to the metabolism of glyceraldehyde-3-phosphate to pyruvate in tissues from rats (Veech et al., 1979).

Metabolite	Erythrocytes*	Rat brain	Rat liver	Rat muscle
Lactate/Pyruvate	13.5	13.2	5.3	9.73
[ATP]/[ADP]_f_[P_i_] M^–1^	5,700*	30,000	16,300	27,200
[PEP] μM	33	8	173	17
[ATP] mM	2.25	2.59	3.38	8.05
[P_i_] mM	1.65	2.72	4.76	8.00
[ADP]_f_ μM	248	32	46	37
[CrP] mM	–	4.72	–	26.6
[Cr]	–	6.11	–	12.8
[DHAP] μM	17	19	43	17
*[G-3-P]*μ*M*	*3.7*	*4.2*	*9.5*	*3.7*
[NADH]/[NAD^+^]	0.0025	0.0024	0.00098	0.0018
*[PEP]/[G-3-P]*	*8.9*	*1.9*	*18.2*	*8.9*
K* (M^–1^)	*126*	*136*	*290*	*435*
ΔG_ATP_ Kcal/mole	−13.65 ± 0.07	−14.08 ± 0.01	−14.03 ± 0.08	−13.69 ± 0.06

Experimental data reported by Veech et al. (1979) for tissues from rats. The equilibrium constant for triosphosphate isomerase was assumed to be 0.22 and [G-3-P]_f_ was calculated from the measured [DHAP]. [NADH]/[NAD^+^] was calculated from the lactate/pyruvate ratio assuming a lactate dehydrogenase equilibrium constant at 37°C and pH 7.2 of 1.85 × 10^–4^ M^–1^ (Williamson et al., 1967). *Erythrocytes have a lower [Mg^2+^] (0.15 mM vs 1 mM) and mature erythrocytes are not living (can not reproduce). They are not included in most of the discussion. Metabolite concentrations/ratios in italics were calculated from the data of Veech et al. (1979) but were not explicitly presented in their paper.

This correction for bound ADP is large and its measurement significantly advanced the understanding energy metabolism. Similar questions were raised about the extent of binding of ATP and Pi. This has been largely answered by ^31^P MRI measurements in tissues, both perfused and *in vivo* (Iles et al., 1985; Barstow et al., 1994; McCreary et al., 1996; Haseler et al., 1998, 1999). The [ATP] concentration measured by ^31^P MRI are essentially the same as that measured by total chemical analysis of the tissues, consistent with most of the ATP being free in solution. On the other hand, ^31^P MRI measurements in perfused liver, for example, were consistent with only about 1/3 of the chemically measured Pi being free in solution (Iles et al., 1985). Measurements in skeletal muscle *in vivo* by ^31^P MRI suggest that a similar fraction is bound in resting muscle (Barstow et al., 1994; McCreary et al., 1996; Haseler et al., 1998, 1999). This correction is less than for ADP, but significant, and indicates the physiological set point for energy state and ΔG_ATP_ in living tissues are somewhat higher than calculated by Veech et al. (1979), near 9 × 10^4^ M^–1^ and −62 kJ/mole (−14.8 kcal/mole).

### Influence of Equilibrium of Reactions 1–4 on the Kinetic Behavior of Reaction 5 (Pyruvate Kinase)

Because the reactions from G-3-P to PEP are near equilibrium, an overall expression can be written that relates [G-3-P] to the concentration of PEP:

(6) $${\text{K}}^{*}=\left(\left[\text{PEP}\right]/\left[\text{G-}\,3\,\text{-P}\right]\right)\,\times \,\left(\left[\text{NADH}\right]/\left[{\text{NAD}}^{+}\right]\right)\,\times \,\left(\left[\text{ATP}\right]/{\left[\text{ADP}\right]}_{\text{f}}\,\left[\text{Pi}\right]\right)$$

where K^∗^ is the product of the equilibrium constants for the included reactions (Eqs 1–4). The value for K^∗^ can be calculated from measured metabolite concentrations; [G-3-P], [PEP], cytoplasmic [NADH]/[NAD^+^], and [ATP]/[ADP]_f_[Pi] from the measured metabolite concentrations shown in Table 1 (Veech et al., 1979). The values for K^∗^ calculated for brain, liver, and muscle, are 136, 290, and 435 M^–1^, respectively. As expected for the reactions being near equilibrium in each tissue, the values for K^∗^ are not significantly different. Solving for [PEP]:

(7) $$\left[\text{PEP}\right]={\text{K}}^{*}\,\times \,\left[\text{G-}\,3\,\text{-P}\right]\,\times \,{\left(\left[\text{NADH}\right]/\left[{\text{NAD}}^{+}\right]\right)}^{-1}\times \,{\left(\left[\text{ATP}\right]/{\left[\text{ADP}\right]}_{\text{f}}\,\left[\text{Pi}\right]\right)}^{-1}$$

As seen from Eqs (6, 7), the concentrations of both substrates for PK are dependent on the energy state and [NADH]/[NAD^+^]. Decrease in energy state, assuming no change in [G-3-P] and [NADH]/[NAD^+^], increases the concentrations of PEP and ADP. Thus, decrease in energy state is associated with an increase in the concentrations of both substrates for PK, [ADP]_f_, and [PEP].

#### Quantification of the Dependence of Flux Through PK on Near Equilibrium Reactions 1–4: A. the Steady State Rate Expression

When acting as an ATP source, the reaction catalized by PK is irreversible and not subject to significant product inhibition (Reynard et al., 1961; Dobson et al., 2002). In order to simulate the behavior under physiological conditions we have assumed the enzyme rapidly equilibrates with its substrates, i.e., a random bi-bi enzyme mechanism:

(8) $$\begin{array}{l} \text{ADP+PK}↔\text{PK-ADP+PEP}⤡\\ \text{PEP+PK}↔\text{PEP-PK+ADP}⤢ \end{array}$$

PEP-PK-ADP⟶Pyr+ATP

The K_M_ values for the substrates were also assumed to be equal to the dissociation constants. At steady state the rate for production of ATP or pyruvate (v) can be expressed:

(9) $$1/\text{v}=1/\text{Vm}+{\text{K}}_{\text{Madp}}/\left(\text{Vm}\times \left[\text{ADP}\right]\right)+{\text{K}}_{\text{Mpep}}/\left(\text{Vm}\times \,\left[\text{PEP}\right]\right)+{\text{K}}_{\text{Mpep}}\times K_{\text{Madp}}/\left({\text{V}}_{\text{m}}\times \,{\left[\text{ADP}\right]}_{\text{f}}\,\left[\text{PEP}\right]\right)$$

where K_M_^adp^ and K_M_^pep^ are the Michaelis constants for ADP and PEP, respectively and Vm is the maximal activity of PK as measured at saturation with both substrates. Eq. (7) can be used to solve for [PEP] at any value for [ADP]_f_ and [NADH]/[NAD^+^] and flux through PK calculated from Eq. (9). The rate can be expressed as production of either product (ATP or pyruvate) and ATP production was chosen to emphasize the role in energy metabolism. Since the simulations are for steady states, the rate of ATP production is the same as the rate of ATP consumption. When other sources of ATP are present, such as oxidative phosphorylation, the simulations apply only to ATP produced by PK.

#### Quantification of the Dependence of Flux Through PK on the Near Equilibrium Reactions 1–4: B. Simulation of PK Activity for Physiological Conditions Using MatLab^1^

Flux through PK (Eq. 9) is dependent on several metabolic variables, [G-3-P], [NADH]/[NAD^+^], [ATP], [ADP]_f_, [Pi], K_M_^adp^, K_M_^pep^, and Vm for PK. Table 2 lists the values of metabolic parameters used to simulate flux through PK. In order to be consistent with function *in vivo*, the total adenine nucleotide ([ATP] + [ADP]_f_) and creatine ([CrP] + [Cr], muscle only), concentrations were maintained constant and the creatine kinase reaction ([ATP][Cr]/[CrP][ADP], Keq = 140) at equilibrium. The total concentrations of adenine nucleotides and creatine were set for resting conditions (Veech et al., 1979), and [ADP]_f_ used in all calculations. The phosphate pool was maintained constant (i.e., [Pi] + [ATP] + [CrP] = constant). Conversion of ATP to ADP and of creatine phosphate to creatine are associated with stoichiometric increase in [Pi]. Resting [Pi] values of 3.4 and 4 mM were used for liver and muscle, respectively. These are lower than the measured values (4.76 and 8 mM) in recognition that chemical analysis in tissue samples normally gives higher values than *in vivo* ^31^P NMR. The presented simulations have been limited to skeletal muscle and liver because they are tissues with the greatest metabolic differences (with and without creatine kinase and high total creatine). Experimental values for Vm = 387 and 50 μmoles/min/g wet weight and [ATP] = 8 and 3.4 mM for resting skeletal muscle and liver, respectively (Veech et al., 1979), have been used in the calculations. The values of K_M^adp_ and K_M^pep_ were assumed to be 300 and 400 μM. The simulations were carried out for the range in [ADP]_f_ observed under normal physiological function. In skeletal muscle, for example, a change in [ADP]_f_ from 27 to 115 μM is typical for the transition between rest and moderate work. This transition is associated with an increase in the rate of ATP consumption of 20–50 fold and a decrease in [CrP]/[Cr] from 2 to 0.5 (McCreary et al., 1996; Haseler et al., 1998, 1999; Wilson, 2015a, b, 2016, 2017; Wilson and Matschinsky, 2018).

**TABLE 2:** Metabolic parameters used to simulate flux through PK.

Parameter	Liver (resting)	Skeletal muscle (resting)
K_M_^adp^	300 μM	300 μM
K_M_^pep^	400 μM	400 μM
Vm	50 μmol/min/g tissue	387 μmol/min/g tissue
[G-3-P]	8 μM	8 μM
[ATP]	3.4 mM	8 mM
[CrP] + [Cr]	0	40 mM
[Pi]	2 mM	4 mM
K*	800 M^–1^	800 M^–1^
[ATP]/[ADP]_f_[Pi]	37,000 M^–1^	54,000 M^–1^

*The simulations for flux through PK were based on the parameter values listed in this table. The values are for resting tissue and [Pi] has been decreased (4.76 to 2 in liver; 8 to 4 in skeletal muscle) to partially correct for binding as indicated by ^31^P MRI (Iles et al., 1985).*

#### The Relationship of [ADP]_f_ to the Rate of Production of ATP by PK

As noted earlier, flux through PK is determined by the rate of ATP consumption, and it is important to understand the metabolic basis for coupling of consumption to production. Increase in ATP consumption results in a decrease in [ATP] and, more importantly, increase in [ADP]_f_ and [Pi]. This decreases the energy state and, as indicated by eq. (9), increases the concentrations of both substrates for PK. Increase in its substrate concentrations increases flux through PK and the rate of ATP production until ATP production again equals consumption, but at a lower energy state, for as long as moderate exercise continues. In most cells, [ATP] and [Pi] are at least a factor of 30 larger than [ADP]_f_, and latter is primarily responsible for the change in energy state. The presence of creatine kinase and high concentrations of Cr and CrP, as in skeletal muscle, increases complexity of the metabolic response. In cells with creatine kinase, decrease in [ATP]/[ADP]_f_ is accompanied by decrease in [CrP]/[Cr] through equilibration of creatine kinase. When the total creatine concentration ([Cr] plus [CrP]) is high, as in skeletal muscle, the change in [Pi] makes a substantial contribution to the change in energy state.

**FIGURE 3:**
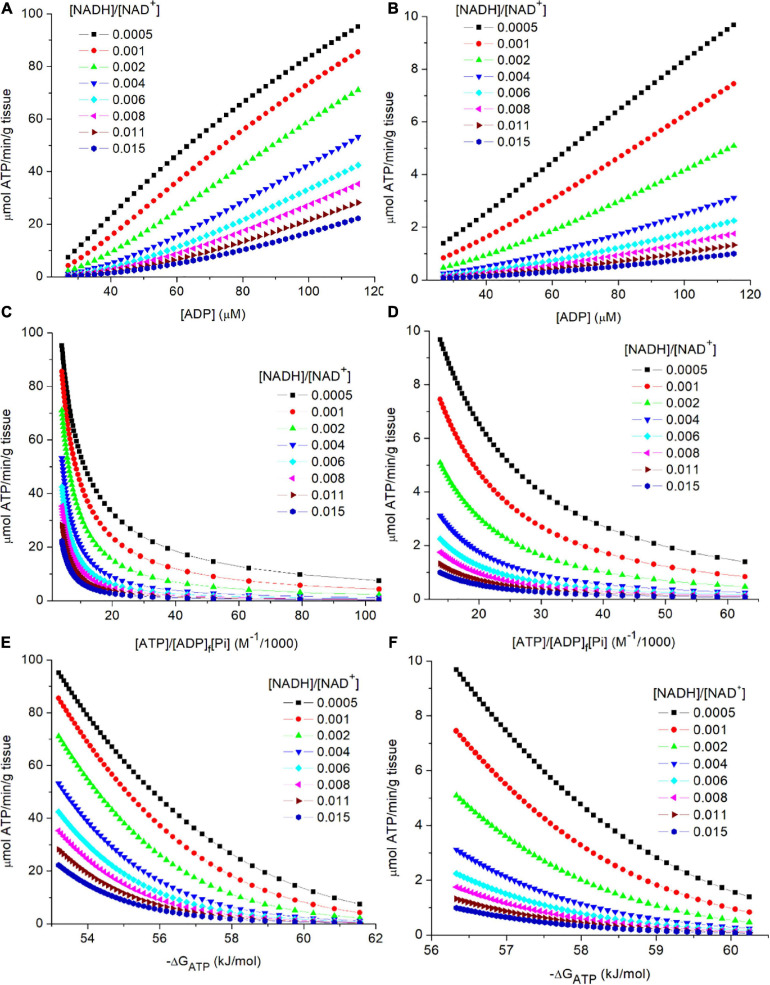
**(A–F)** Simulation of the relationship of flux through PK to the metabolic variables in the preceding near equilibrium reactions. As described in the text, PK was assumed to be irreversible and to equilibrate with its substrates in a random bi bi enzyme mechanism without product inhibition. The steady state rate expression (see text equation 9). The simulated concentrations and rates are shown for 2 tissues, skeletal muscle **(A,C,E)** and liver **(B,D,F)**. The variables associated with PK (K_M_adp, K_M_pep, and the Vm) were assumed tissue specific and constant. Experimental values for [ATP] and Vm (Veech et al., 1979) were used: Vm = 387 and 50 μmoles/min/g wet weight and [ATP] = 8and 3.4 mM for skeletal muscle and liver, respectively. Simulations for skeletal muscle, but not liver, included equilibrium of the creatine kinase reaction. The simulated range in [ADP]_f_ (27 to 115 μM) corresponds to a range in [CrP]/[Cr] of 2 to 0.5. [G-3-P] was assumed to be constant at 8 μM, and [Pi] was set to be near 4 mM when the [CrP]/[Cr] was near 2 (resting muscle). Note that [PEP] is proportional to [G-3-P], other variables held constant, so the dependence on [G-3-P] s not presented. The simulations were carried out using MatLab (www.mathworks.com). **(A,B)** presents the relationship of flux through PK to [ADP]_f_ for cytoplasmic [NADH]/[NAD^+^] values from 0.0005 to 0.015 for skeletal muscle (3A) and liver (3B). **(C,D)** presents the relationship of flux through PK to the calculated energy state ([ATP]/[ADP]_f_[Pi]) for cytoplasmic [NADH]/[NAD^+^] values from 0.0005 to 0.015. **(E,F)** presents the relationship of flux through PK to the free energy of hydrolysis of ATP (−ΔG_ATP_) at cytoplasmic [NADH]/[NAD^+^] values from 0.0005 to 0.015.

In Figures 3A,B, flux through PK is plotted against [ADP]_f_ for skeletal muscle (3A) and liver (3B). Separate curves are shown for 7 different values for cytoplasmic [NADH]/[NAD^+^]. Increased reduction of the cytoplasmic NAD couple (increase in [NADH]/[NAD^+^]) results in lower flux through PK at each [ADP]_f_). When comparing skeletal muscle to liver, flux in muscle is higher at each [ADP]_f_. As noted earlier, simulations for skeletal muscle included a total creatine pool ([CrP] + [Cr]) of 40 mM (Veech et al., 1979). The creatine kinase reaction was maintained at equilibrium (K_eq_ = [ATP][Cr]/[ADP]_f_[CrP]). As [ATP]/[ADP]_f_ decreases CrP is hydrolyzed to Cr to reduce [CrP]/[Cr] leading to a substantial increase in [Pi]. In skeletal muscle, but not in liver, as [ADP]_f_ increases from 27 to 115 μM, [Pi] increases from 2.8 to 16.9 mM, and [CrP]/[Cr] decreases from 2 to 0.5 noted above. As a result, in skeletal muscle there is a greater decrease in energy state, and increase in flux through PK, than would occur if only [ADP]_f_ had increased. This highlights an important role of creatine kinase in muscle, which is to increase the range in ATP production rates that can be attained without excessive increase in [ADP]_f_, and thereby of [AMP]_f_ (Hardie et al., 2012; Wilson, 2013, 2015a,b, 2017; Hardie, 2015; Wilson and Matschinsky, 2018).

#### Relationship of the Rate of ATP Synthesis by PK to [ATP]/[ADP]_f_[Pi] and the Free Energy of Hydrolysis of ATP

As is evident in eq. 9, flux through PK is not determined by individual concentrations of ATP, ADP, and Pi but by the reaction equilibria. In Figures 3C,D, flux through PK is plotted against the energy state ([ATP]/[ADP][Pi]) as appropriate for the contribution of concentration of each reactant to equilibrium. The range in energy state for muscle is larger than for liver, consistent with the larger range in flux through PK (≈40x vs ≈6x) for the tissues *in vivo*. Although the range in [ADP] used for simulating muscle and liver were the same, the change in [Pi] due to the changes in [CrP] in muscle, but not liver, results in a larger change in energy state in muscle. This is also observed when the rate through PK is plotted against the free energy of hydrolysis for the terminal phosphate of ATP (−ΔG_ATP_) as seen in Figures 3E,F. For the simulations, [G-3-P] was held constant in order to focus attention on the roles of energy state and cytoplasmic [NADH]/[NAD^+^]. At each ATP consumption rate, as energy state increases cytoplasmic [NADH]/[NAD^+^] become more oxidized in order to sustain that flux. As emphasized earlier, flux through PK is determined by the rate of ATP consumption. Changes in ATP consumption alter the energy state and thereby metabolite concentrations within reactions 1–4. Energy state induced changes in [ADP]_f_ and [PEP] alter the rate of ATP synthesis by PK as appropriate for attaining a new steady state in which synthesis again equals consumption. One way to view the result is that since ADP is a required substrate, the PK flux cannot change unless there is a change in ADP production (ATP consumption), i.e., changes in [G-3-P], [NADH]/[NAD^+^], etc. alter the mass action ratios but not the flux. Flux is strictly coupled to ATP consumption.

#### Relationship of the Activity of PK to the Concentration of AMP, an Important and Early Regulator of Energy Metabolism

In order to establish a set point for energy metabolism it is necessary for reactions 1–5 to not only establish an auto-regulated set point but also to provide effective communication of that set point to the rest of metabolism. This was accomplished in part through the presence of adenylate kinase, another early, and near equilibrium enzyme:

(10) 2⁢A⁢D⁢P=ATP+AMP   Keq= 1

**FIGURE 4:**
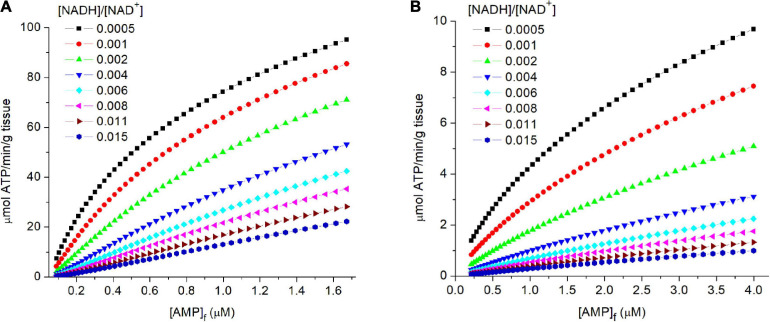
**(A,B)** The relationship of flux through PK to the levels of free AMP ([AMP]_f_) in tissue. In the simulations described in the legend of Figure 3, the concentrations of free AMP were calculated assuming equilibration of adenylate kinase and an equilibrium constant of 1. The calculated flux through PK is plotted against [AMP]_f_ (abscissa). The [AMP]_f_ concentration for 50% activity of AMP dependent protein kinase (AMPK) in isolated perfused rat heart has been reported to be 1.8 μM (Frederich and Balschi, 2002).

Figures 4A,B shows the relationship of [AMP]_f_ to flux through PK for both muscle (Figure 4A) and liver (Figure 4B). Equilibration of adenylate kinase, combined with a relatively high and constant [ATP], results in the concentration of free AMP ([AMP]_f_) varying as the square of [ADP]_f_: i.e., [AMP]_f_ = [ADP]_f_^2^/[ATP] where [ATP] is essentially constant. The great sensitivity of [AMP]_f_ to changes in the energy state made it an ideal small molecule “messenger,” rapidly distributing throughout the cell and providing accurate and sensitive measure of the homeostatic set point. Evolution extended and amplified the role of AMP in regulating energy metabolism through an array of added mechanisms dependent on [AMP]_f_. This includes AMP dependent protein kinase (AMPK), a key regulator of energy metabolism (Hardie et al., 2012; Mihaylova and Shaw, 2012; Hardie, 2015; Wilson and Matschinsky, 2019). Frederich and Balschi (2002) determined the [AMP]_f_ concentration for half maximal activity of AMPK in perfused rat heart and obained a value of 1.8 μM, consistent with the simulated [AMP]_f_ for physiological metabolic rates in both skeletal muscle and liver.

#### The Influence of Cytoplasmic [NADH]/[NAD^+^] on Flux Through PK

The contribution of cytoplasmic [NADH]/[NAD^+^] in determining flux through PK is illustrated in each panel of Figures 3, 4. For any selected metabolic parameter plotted on the abscissa ([ADP], energy state ([ATP]/[ADP]_f_[Pi]), or ΔG_ATP_), the rate of ATP synthesis increases with decrease in [NADH]/[NAD^+^]. Simulations are presented for [NADH]/[NAD^+^] ranging from 0.0005 to 0.015. This range corresponds to lactate/pyruvate ratios from 2.7 to 81, encompassing the full range of values observed *in vivo* (Williamson et al., 1967; Christensen et al., 2014). The cytoplasmic [NADH]/[NAD^+^] calculated from the [lactate]/[pyruvate] reported by Veech et al. (1979) for each tissue are included in Table 2. This inhibition of flux through PK by increasing reduction of cytoplasmic NAD is not sufficiently appreciated. Under physiological conditions increase in lactate/pyruvate, indicating decrease in [NADH]/[NAD^+^], most often occurs when the energy state is decreasing. Decrease in energy state counters (masks) the effect of increase in [NADH]/[NAD^+^]. This is not always the case, however. The interactions of cytoplasmic [NADH]/[NAD^+^], energy state, and flux through PK need to be kept in mind when alterations in lactate/pyruvate or inhibition of lactate dehydrogenase are involved (Sola-Penna, 2008; Lea et al., 2010; Leite et al., 2011; Christensen et al., 2014). As will be discussed later, [NADH]/[NAD^+^] plays an important role in determining the compatability of oxidative and fermentive metabolism.

#### What Is the Role of PK in Regulating the Rate of ATP Synthesis?

The Vm of PK primarily determines the maximal rate of ATP synthesis that can be attained through the pathway. PK activity also contributes to determining the energy state and [NADH]/[NAD^+^] at which any particular rate of ATP production can be sustained. Doubling the maximal activity (Vm) for PK, for example, with no change in ATP consumption would have no effect on the steady state flux through PK. Instead it would lead to a small increase in the energy state (decrease in [PEP] and [ADP]_f_) at which that flux was maintained.

## Discussion

A robust and stable source of metabolic energy is essential to life. Once an appropriate energy source was established, as new metabolic pathways were added both the metabolism and its regulation needed to be supportive of/consistent with the existing energy state. All metabolism, be it chemical synthesis (DNA, RNA, protein, and metabolites, etc.), work (ion transport, movement), or metabolic regulation, became coupled to, and supportive of, the set point for energy metabolism. We speculate that the metabolic energy set point was established by reactions 1–5, and this provided the stable platform needed for evolutionary expansion. Metabolism and metabolic regulation became “locked in” to assuring the energy state was maintained at that set point [ΔG_ATP_ near −62 kJ/mole (−14.8 Kcal/mole)]. Reactions 1–4 are present in both archaea and bacteria, and essentially all eukaryotes. It is reasonable to expect that early relatives of modern organisms utilized these reactions and they resulted in the resting energy state being set near 90,000 M^–1^ (ΔG_ATP_ near −62 kJ/mole). This is reminiscent of the view expressed by Kluyver and Donker (1926) that the basic enzymatic reactions which support and maintain life processes within organisms have more similarities than differences, “Die Einheit in der Biochemie” (the unity in biochemistry). This unity can be difficult to see, particularly when metabolism of extremophiles, organisms living as obligate anaerobes or at extreme temperatures or pH, is included. Extremophiles often use quite different chemistry and/or reaction mechanisms in order to accommodate stress imposed by the environment (Fuchs, 1989, 2011; Ronimus and Morgan, 2003; Tabita et al., 2008; Berg et al., 2010; Berg, 2011; Edirisinghe et al., 2016; Mangiapia and Scott, 2016; Zhang et al., 2017; Oren, 2020). It should be kept in mind that once early prokaryotes (bacteria, archaea) had formed, they reproduced by cell division. As a result, they were never without a full complement of enzymes and metabolites. Having pre-existing enzymes and metabolites would increase the possibility of replacing environmentally compromised metabolic processes with ones more suited to the new environment. This raises questions about where and how the first life forms developed; did an extremophile develop first and then adapt to more “normal” environments or the reverse. Evolution proceded in both directions and that makes it difficult to establish the characteristics of the earliest organisms.

### Integration of Early Fermentative Metabolism With Oxidative Phosphorylation

Eukaryotes are thought to have formed through archaea internalizing a symbiotic bacterium that had a fully developed respiratory chain and oxidative phosphorylation (Margulis, 1970; Tabita et al., 2008; Gray, 2012; Eme et al., 2017). Reasonable speculation on the identity of the internalized symbiote has centered on the α-proteobacterium, *P. denitrificans*, and its relatives (Kornberg et al., 1960; Margulis, 1970; John and Whatley, 1975, 1977; Kristjansson et al., 1978; Erecińska et al., 1979; Meinhardt et al., 1987; Andersson et al., 1998; Yip et al., 2011; Gray, 2012; Eme et al., 2017). Aerobically grown *P. denitrificans* has a fully developed respiratory chain with phosphorylation and regulatory properties similar to those in mitochondria. Comparison of iron-sulfur components of the first site of oxidative phosphorylation (NADH:ubiquinone oxidoreductase or Complex 1) by Meinhardt et al. (1987) indicated these are very similar to those in mammalian mitochondria. Yip et al. (2011) reported that in addition to the 14 core subunits, the complex also contains homologues of three supernumerary mitochondrial subunits: B17.2, AQDQ/18, and 13 kDa (originally thought to be added in eukaryotes, bovine nomenclature). The remarkable biochemical and genetic similarities of mitochondrial oxidative phosphorylation in eukaryotes to that in *P. denitrificans* are consistent with mitochondria having arisen from an endocytosed α-proteobacteria closely related to *P. denitrificans*. Symbiosis was facilitated by the two organisms having metabolism coupled to the same set point for energy metabolism and, therefore, many compatible regulatory mechanisms. As an important negative, these common regulatory mechanisms also facilitated the development of bacterial parasites, such as *Rickettsia prowazekii* (Andersson et al., 1998). In the case of mitochondria, the endocytosed prokaryote was metabolically integrated and eukaryotes with mitochondria and oxidative phosphorylation established by 1 Gya when the rise in atmospheric oxygen triggered the Cambrian phylogenetic “explosion.” This was a relatively brief, in geological time, period in which a large number of different phyla appeared, including those giving rise to all higher plants and animals. Increased atmospheric oxygen was permissive for developing specialized cells and structures by differentiation through increasing the amount of ATP that could be synthesized from organic fuels. Glucose provides 1 or 2 ATP/glucose from the Entner-Doudoroff and Embden-Meyerhof-Parnas pathways, respectively, and adding oxidative phosphorylation increased the yields to 32–38 ATP/glucose. In addition, the waste products of oxidative phosphorylation are water and CO_2_ and not the organic acids produced by fermentation. Eukaryotes with mitochondria were uniquely positioned to take advantage of the increase in atmospheric oxygen at the beginning of the Cambrian period. The combination of a large increase in available metabolic energy and readily eliminated waste products, water and CO_2_, made possible evolutionary development of higher plants and animals.

Formation of mitochondria from endocytosed prokaryotes introduced an interesting metabolic mismatch. Oxidative phosphorylation developed in chemoautotropes, which include nitrogen fixing bacteria located in the soil, iron oxidizing bacteria located in lava beds, and sulfur oxidizing bacteria located in deep sea thermal vents. Chemoautotropes derive metabolic energy from oxidation/reduction reactions using inorganic metabolites in the environment. As noted earlier, the precursors for mitochondria are believed to have been bacteria that developed oxidative phosphorylation using nitrate as the chemical oxidizer. Until oxygen became available, the electron transport chain in *Nitrobacte*r consisted of only the first two sites, NADH to cytochrome c, and cytochrome c was oxidized by nitrate. Note: *p. denitrificans* also has a soluble copper protein, pseudoazurin, with the same half reduction potential as cytochrome c, that can carry out the same oxidation-reduction reactions (Kukimoto et al., 1995; Pearson et al., 2003). The first two steps of nitrate reduction are catalized by nitrate reductase, a molybdoenzyme(s) that reduces nitrate (NO_3_^–1^) to nitrite (NO_2_^–1^), and nitrite reductase, an enzyme that reduces nitrite to nitric oxide (NO; Berks et al., 1995; Pearson et al., 2003).

(11) $${\text{NO}}_{3}^{-1}+2\,H^{+}+2\,e^{-}={\text{NO}}_{2}^{-1}+{\text{H}}_{2}\,\text{O}$$

Eo=′ 0.42V

(12) $${\text{NO}}_{2}^{-1}+2\,H^{+}+2\,e^{-}=\text{NO}+{\text{H}}_{2}\,\text{O}$$

Eo=′ 0.37V

In order to have sufficient energy for synthesis of 2 ATP (ΔE = 2 × 0.32 V) for each NADH oxidized (Sites 1 plus 2), the redox potential difference between NAD (E^o^’ = −0.32 V) and both nitrate reduction to nitrite (E^o^’ = +0.42 V) and nitrite reduction to NO (Em = 0.37 V) needs to be greater than 0.64 V. Additional energy loss is needed to make the cytochrome c to NO_3_^–^ reaction irreversible and allow control, and this adds at least 0.1 V (i.e., 0.64 V + 0.1 V = 0.74 V). To make ATP by Sites 1 and 2 of the respiratory chain with nitrate as the electron acceptor, the potential of the NAD couple needs to be near −0.34 V (+0.4 V + −0.74 V = −0.34 V; [NADH]/[NAD^+^] ≈ 2). Although *P. denitrificans* can grow on glucose, it can not use reactions 1–5 (glycolysis) for energy production. At physiological energy states, the high [NADH]/[NAD^+^] required for oxidative phosphorylation means the equilibrium concentration of PEP is very low and reactions 1–4 function in the direction of gluconeogenesis, i.e., [PEP] is reduced and used for biosynthesis. Glucose has to be catabolized using the less energy efficient Entner-Duderoff and pentose phosphate pathways (John and Whatley, 1975, 1977; Dunstan et al., 1984). Thus, as for mitochondria in eukaryotes (Williamson et al., 1967), the [NADH]/[NAD^+^] ratio in *P. denitrificans is* approximately 1000 times more reduced than in the cytoplasm of eukaryotes where reactions 1–5 were used to make ATP ([NADH]/[NAD^+^] ≈2 vs ≈0.002).

As oxygen became more available in the environment (in and under mats of blue green cyanobacteria?) and the concentrations slowly increased, oxidation of cytochrome c by nitrate was replaced with oxidation by molecular oxygen. This occurred in steps, first through an oxidase with a high affinity for oxygen that could functionally replace cytochrome c oxidation by nitrate reductase and then, with further increase in oxygen, addition of the third coupling site (cytochromes a,a_3_) to the electron transport chain. This metabolic transition was able to occur despite very low atmospheric pO_2_ because local pO_2_ in, and immediately below, growing mats of blue green cyanobacteria substantially exceeded the atmospheric pO_2_. It can be speculated that it was in local environments with higher than atmospheric pO_2_ that a *P. denitrificans* ancestor developed and was endocytosed, resulting in eukaryotes with mitochondria. As a result of the differences in [NADH]/NAD^+^] between the host and endocytosed bacterium, only part of the metabolism of the two organisms could merge. Metabolism essential to oxidative phosphorylation; citric acid cycle, and fatty acid oxidation, etc., remained separated from the cytoplasm by a membrane that prevented NADH and NAD^+^ from moving from one compartment to the other. Greater reduction of NAD, essential to oxidative phosphorylation and metabolism in the bacterium, would inactivate reactions 1–5, disrupting metabolism in the host. The newly formed eukaryotes would likely have been restricted to living in or under mats of cyanobacteria until the increase in atmospheric pO_2_ that occurred at the beginning of the Cambrian period. Compartmentation allowed the metabolism of organisms that had adapted to the same energy state, but different [NADH]/[NAD^+^] levels, to be functionally integrated.

1. Living organisms require energy for their existence and life, as we know it, is based on, and regulated by, thermodynamics. Thermodynamic control is exerted through near equilibrium reactions that determine the concentrations of metabolic substrates for enzymes that catalyze irreversible steps in the metabolic pathways and/or act to regulate the activity of those enzymes through control of the concentrations of regulatory molecules (AMP, ADP, etc.). As a result, metabolism has a robust, thermodynamically determined, set point (−ΔG_ATP_) and long term (virtually unlimited) stability. The rest of metabolism and its regulation is constrained to maintain this set point (homeostasis).
2. Metabolic regulation based on thermodynamics is different from that based on kinetics. This is important because thermodynamic parameters are stable whereas kinetic parameters are highly variable. This is clear when describing the ATP producing part of the Embden-Meyerhof-Parnas pathway. The kinetic viewpoint focuses on the two irreversible reactions, phosphofructokinase (PFK) and PK. The steady state flux (ATP synthesis) by the pathway is necessarily equal to the flux through PK and PFK, although the latter only assuming no significant loss or gain of metabolites prior to PK. It is argued, therefore, PK and PFK control flux through the pathway. Although the observation is correct, the conclusion is misleading. ATP producing flux through PK is primarily determined by the rate of ATP consumption as it effects reactions 1–4, i.e., flux is primarily determined by the near equilibrium reactions between PFK and PK. As the rate of ATP consumption changes, the induced mismatch between use and production leads to alterations in energy state and this changes [ADP]_f_ and [PEP]. The changes in [ADP]_f_ and [PEP] alter flux through PK appropriately for restoring equality of ATP production and consumption. Changes in energy state also provide for feedback regulation of PFK, largely through the allosteric regulator [AMP]_f_, that maintains supply of G-3-P.
3. As evolution added more metabolism, further layers of control were needed to integrate the new reactions into existing metabolism without changing the homeostatic set point. Addition of gluconeogenesis, for example, resulted in regulation to PK and PFK to prevent futile cycling, i.e., PK and PFK need to be turned off during gluconeogenesis because flux through either enzyme would waste energy (ATP), subtracting from net glucose synthesis, and decreasing overall efficiency.

## Data Availability Statement

All figure stimulations presented in this study are included in the article/supplementary material.

## Author Contributions

DW was primarily responsible for writing the manuscript with continuous editing/correction by FM. Both authors contributed to the article and approved the submitted version.

## Conflict of Interest

The authors declare that the research was conducted in the absence of any commercial or financial relationships that could be construed as a potential conflict of interest.

## Footnotes

1 www.mathworks.com
